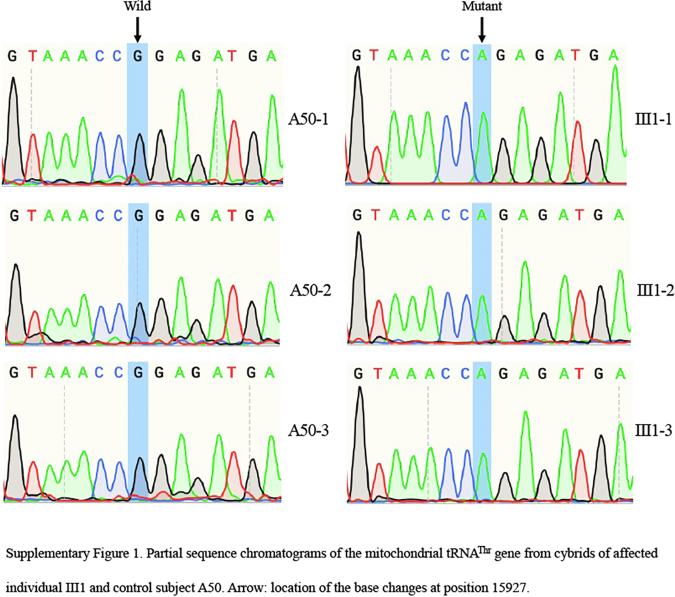# Defective post-transcriptional modification of tRNA disrupts mitochondrial homeostasis in Leber’s hereditary optic neuropathy

**DOI:** 10.1016/j.jbc.2024.107728

**Published:** 2024-08-28

**Authors:** Juanjuan Zhang, Wenxu Li, Zhen liu, Yingqi Chen, Xiaoyang Wei, Lu Peng, Man Xu, Yanchun Ji

**Affiliations:** 1 State Key Laboratory of Ophthalmology, Optometry and Vision Science, Eye Hospital, Wenzhou Medical University, Wenzhou, Zhejiang, China; 2 Attardi Institute of Mitochondrial Biomedicine, School of Laboratory Medicine and Life Sciences, Wenzhou Medical University, Wenzhou, Zhejiang, China; 3 Institute of Genetics, Zhejiang University School of Medicine, Hangzhou, Zhejiang, China

**Keywords:** Leber’s hereditary optic neuropathy (LHON), mitochondrial tRNA^Thr^ mutation, autophagy, mitophagy, apoptosis

## Abstract

Leber's Hereditary Optic Neuropathy (LHON) is a rare, maternally inherited eye disease, predominantly due to the degeneration of retinal ganglion cells (RGCs). It is associated with a mitochondrial DNA (mtDNA) point mutation. Our previous study identified that the m.15927G > A homoplasmic mutation damaged the highly conserved base pairing (28C-42G) in anticodon stem of tRNA^Thr^, caused deficient t^6^A modification and significantly decreased efficiency in aminoacylation and steady-state levels of tRNA^Thr^, and led to mitochondrial dysfunction. Meanwhile, mechanisms underlying mtDNA mutations regulate intracellular signaling related to mitochondrial and cellular integrity are less explored. Here, we manifested that defective nucleotide modification induced by the m.15927G > A mutation interfered with the expression of nuclear genes involved in cytoplasmic proteins essential for oxidative phosphorylation system (OXPHOS), thereby impacting the assemble and integrity of OXPHOS complexes. As a result of these mitochondrial dysfunctions, there was an imbalance in mitochondrial dynamics, particularly distinguished by an increased occurrence of mitochondrial fission. Excessive fission compromised the autophagy process, including the initiation phase, formation, and maturation of autophagosomes. Both Parkin-mediated mitophagy and receptor-dependent mitophagy were significantly impaired in cybrids haboring the m.15927G > A mutation. These changes facilitated intrinsic apoptosis, as indicated by increased cytochrome c release and elevated levels of apoptosis-associated proteins (*e.g.*, BAK, BAX, cleaved caspase 9, cleaved caspase 3, and cleaved PARP) in the mutant cybrids. This study demonstrates that the m.15927G > A mutation contributes to LHON by dysregulating OXPHOS biogenesis, aberrant quality control, increased autophagy, inhibited mitophagy, and abnormal apoptosis.

Leber's hereditary optic neuropathy (LHON, MIM#535000) is an infrequent ocular condition that follows a maternally inherited pattern, resulting from mutations in multiple genes encoded by mitochondrial DNA (mtDNA) (1, 2, 3, 4). Mitochondria, known for energy generation *via* the oxidative phosphorylation system (OXPHOS), also play a crucial role in mediating cellular signals (5, 6, 7). Mitochondrial damages are linked to various clinical abnormalities (5). Cellular mitochondrial health is maintained through biogenesis, dynamic fusion, fission, and targeted degradation processes (8, 9). Mitochondrial biogenesis is a coordinated process involving mitochondrial genes, which encode 13 polypeptides for the OXPHOS, 2 rRNAs, and 22 tRNAs. The 13 OXPHOS subunits encoded by mtDNA are produced by a mitochondrial translation machinery consisting of 22 tRNAs, 2 rRNAs, and nucleus-encoded constituents such as ribosomal proteins and mitochondrial translation elongation factor (TUFM) (10, 11). Nuclear genes also play a crucial role in this process. They encode over 1500 proteins found in the mitochondrion, comprising 72 OXPHOS system subunits. These subunits are formed in the cytosol and subsequently translocated to the mitochondria (12, 13). To maintain mitochondrial quality control through mitochondrial dynamics (fusion/fission) and mitophagy, mitochondria have defense pathways to respond to intrinsic and extrinsic stressors (14, 15, 16). Mutations in tRNA can cause mitochondrial dysfunction, which can then disrupt nuclear gene expression and impact cellular processes including autophagy and apoptosis through mitochondrial retrograde signaling pathways (6). However, the processes by which deficient tRNA post-transcriptional changes govern the biogenesis of OXPHOS, mitochondrial quality controls, autophagy, and apoptosis are still understudied.

The m.15927G > A mutation affected a highly conserved guanine at position 42 at the anticodon-stem of tRNA^Thr^, destabilizing the conservative base pairing (28C-42G). It resulted in unstable tRNA^Thr^ structure that was supported by decreased melting temperature and slower electrophoretic mobility of mutated tRNA, and deficient N^6^-threonylcarbamoyladenosine (t^6^A) modification of tRNA^Thr^ (17). LHON-associated m.15927G > A mutation has been demonstrated to lower the steady-state levels of mitochondrial tRNA^Thr^, disrupt mitochondrial translation, decrease mitochondrial ATP levels, and enhance reactive oxygen species (ROS) production (18). This mutation-induced impairment may also influence the expression of nuclear-encoded mitochondrial proteins involved in mitochondrial biogenesis, autophagy, and apoptosis. The present investigation reveals that the m.15927G > A mutation disrupts the expression of OXPHOS subunits encoding nuclear genes, thereby affecting the biogenesis and assembly of OXPHOS complexes. The study also investigated whether the dysfunction and instability of OXPHOS, resulting from the m.15927G > A mutation, influence mitochondrial quality control processes, particularly fusion and fission. Furthermore, the study examined whether the disruption of mitochondrial quality control processes affects signaling pathways related to the selectively damaged mitochondria degradation and the intrinsic apoptosis cell death machinery.

## Results

### Dysregulation of nuclear genes encoding subunits of OXPHOS

**Figure 1:**
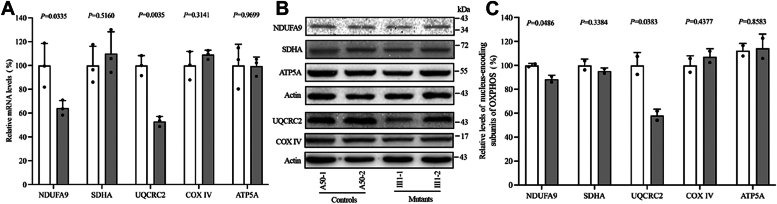
**Decreased mRNA and protein expression levels of CI and CIII subunits.***A*, mRNA expression levels of five OXPHOS subunits (NDUFA9 for CI, SDHA for CII, UQCRC2 for CIII, COX IV for CIV, and ATP5A for CV) in control and mutant groups. *B*, Western blot analysis of five OXPHOS subunits, normalized to Actin. *C*, Quantitative comparison of these subunits in control and mutant cell lines, based on two independent experiments. The comparison of subunits was quantitatively undertaken in control and mutant cell lines. In the graph, the error bars illustrate the standard error of the mean (SEM), and *P* denotes the *t* test significance between control and mutant cybrids.

To explore the impact of the m.15927G > A mutation on OXPHOS biogenesis, we carried out RT-qPCR and Western blot analysis to examine the mRNA and protein levels in 5 subunits of OXPHOS complexes in control and mutant cybrids. These subunits included NDUFA9 (a subunit of NADH dehydrogenase, CI), SDHA (a subunit of succinate dehydrogenase, CII), UQCRC2 (subunit of ubiquinol-cytochrome c reductase, CIII), COX IV (subunit of cytochrome c oxidase, CIV), and ATP5A (subunit of H+-ATPase, CV). Actin served as the loading control. As shown in Figure 1*A*, the mutant cell lines demonstrated significantly decreased mRNA levels in NDUFA9 and UQCRC2 compared to those of the control cell lines. However, the levels of SDHA, COX IV, and ATP5A were comparable to those of the control cell lines. Immunoblotting results indicated an 11.6% reduction in NDUFA9 (*p* = 0.0486) and a 42.1% decrease in UQCRC2 (*p* = 0.0383) in the mutant cells compared to the control cells, while SDHA, COX IV, and ATP5A levels remained comparable (Fig. 1, *B* and *C*).

### Impaired assembly and activity of OXPHOS complexes

**Figure 2:**
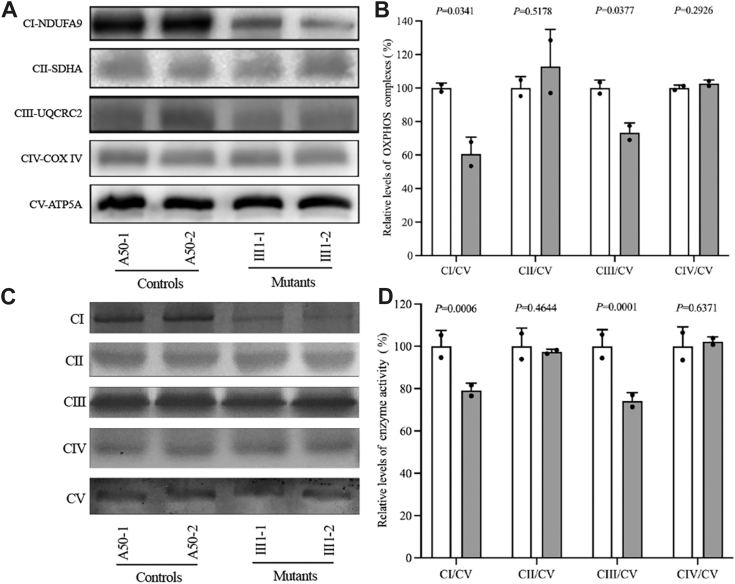
**Assembly and activity of OXPHOS complexes.***A*, BN-PAGE analysis showing steady-state levels of OXPHOS complexes in various cell lines, using antibodies for five subunits (NDUFA9, SDHA, UQCRC2, COX IV, and ATP5A) to assess CI, CII, CIII, CIV, and CV. *B*, quantification of these complexes in control and mutant cell lines. *C*, In-gel activity of CI, CII, CIII, CIV, and CV using specific substrates. *D*, quantitative analysis of in-gel activities for these complexes. Graph details and symbols are explained in Figure 1.

The impact of the m.15927G > A mutation on the assembly and activities of OXPHOS complexes was examined by subjecting the mitochondrial samples to BN-PAGE (19). Cells harboring the m.15927G > A mutation exhibited abnormal assembly of CI and CIII. Particularly, the mean values for CI and CIII were discerned at 60.6% (*p* = 0.0341) and 73.2% (*p* = 0.0337), respectively, within the context of the mutant cell lines relative to the mean values extracted from the control cell lines, as visually depicted in Figure 2, *A* and *B*.

Moreover, CI, CII, CIII, CIV, and CV were assessed for stability and activities using the in-gel activity assay. BN-PAGE was used for separating mitochondrial membrane proteins from various cell lines, and these complexes were then stained with specific substrates. This analysis further verified the impaired assembly of CI and CIII in the mutant cell lines relative to the control cell lines. In particular, the in-gel activities of CI and CIII in mutant cell lines were 79.1% (*p* = 0.0006) and 74.1% (*p* = 0.0001), relative to the average values of control cell lines, respectively (Fig. 2, *C* and *D*). In contrast, the in-gel activities of CII, and CIV in the mutant cell lines were comparable with those of control cell lines.

### Imbalance of mitochondrial dynamics

**Figure 3:**
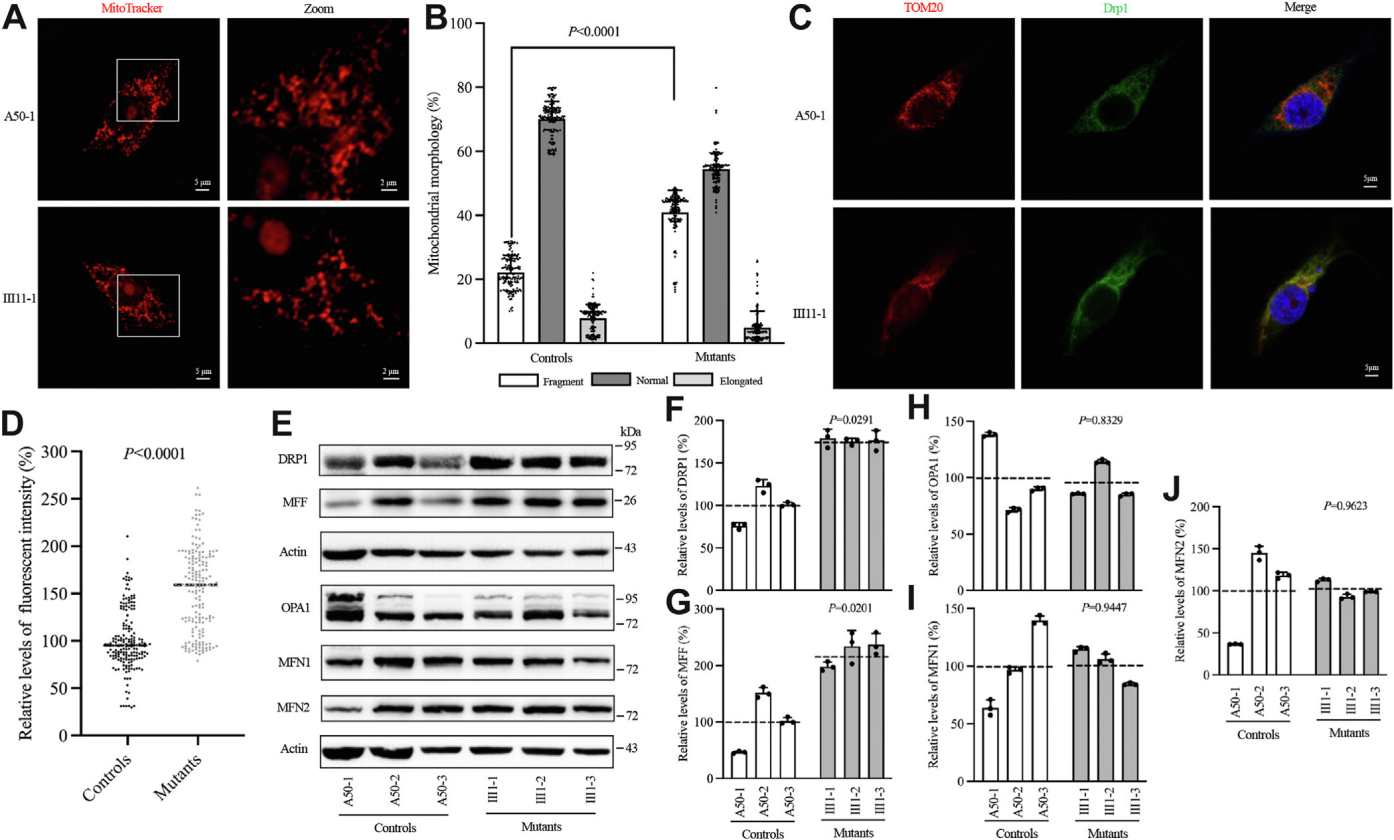
**Impact of m.15927G > A mutation on mitochondrial dynamics.***A*, mitochondrial morphology in control and mutant cybrids visualized using MitoTracker Red dye, analyzed *via* confocal microscopy. *B*, quantification of mitochondrial morphology. Mitochondrial morphology was scored as follows: fragmented, mainly small and round; normal, mixture of round and shorter tubulated; and elongated, long and higher interconnectivity. The percentage of cells with indicated mitochondrial morphologies was determined as a percentage of the total number of cells counted (≥150 cells per experiment). *C*, immunofluorescence examination of DRP1 distribution in control (A50-1) and mutant (III1-1) cybrids using DRP1 antibody labeled with Alex Fluor 488 (green) and MitoTracker, analyzed through confocal microscopy. *D*, quantification of levels of DRP1 fluorescence intensity (≥150 cells per experiment). Three independent determinations were done in each cell line. *E*, utilizing Western blot analysis, we examine the expression of mitochondrial fission-associated proteins (DRP1, MFF) and fusion-associated proteins (OPA1, MFN1, MFN2) across six cell lines, through actin as a loading control. *F-J*, the quantification of these mitochondrial fission- and fusion-associated proteins is detailed. Each cell line was subjected to three independent experiments. Graph details and symbols are explained in Figure 1.

The mitochondrial dysfunctions induced by the m.15927G > A mutation may disrupt mitochondrial homeostasis and integrity, processes sustained through ongoing fission and fusion events. To assess the effect of the m.15927G > A mutation on mitochondrial morphology, Mitotracker Red dye was employed. As shown in Figure 3, *A* and *B*, cybrids harboring the m.15927G > A mutation revealed abnormal mitochondrial morphology, characterized by a significant increase in fragmented mitochondria and a reduction in elongated mitochondrial networks, compared to control cybrids. Dynamin-related protein 1 (DRP1), also called DNM1L (dynamin 1 like), is a dynamin-like GTPase that is an essential modulator of mitochondrial fission. Immunofluorescence staining, using antibodies targeting DRP1 and translocase of outer membrane 20 (TOM20), indicated a 52.8% increase in the levels of DRP1 in the mutant cells compared to the controls, indicating that the mutation causes greater mitochondrial fission (Fig. 3, *C* and *D*).

Moreover, Western blot analysis was employed to determine the levels of fission-related proteins, namely DRP1 and mitochondrial fission factor (MFF), alongside fusion-related proteins, including OPA1 mitochondrial dynamin-like GTPase (OPA1), mitofusin 1 (MFN1), and mitofusin 2 (MFN2), in the two groups. As shown in Figure 3, *E*–*J*, mutant cybrids showed comparable levels of fusion-related proteins but elevating levels of fission-related proteins when compared to controls. Specifically, the average levels of DRP1 and MFF in three mutants were 176.9% (*p* = 0.0291), and 223.2% (*p* = 0.0201) of the average values measured in three control cell lines, respectively (Fig. 3, *E*–*G*). On the other hand, the mutant cybrids' average OPA1, MFN1, and MFN2 levels resembled those of control cells (Fig. 3, *E*, *H*, *I* and *J*). These data suggest that the m.15927G > A mutation resulted in an imbalance in mitochondrial dynamics towards fission.

### Increased autophagy through the ATG pathway

**Figure 4:**
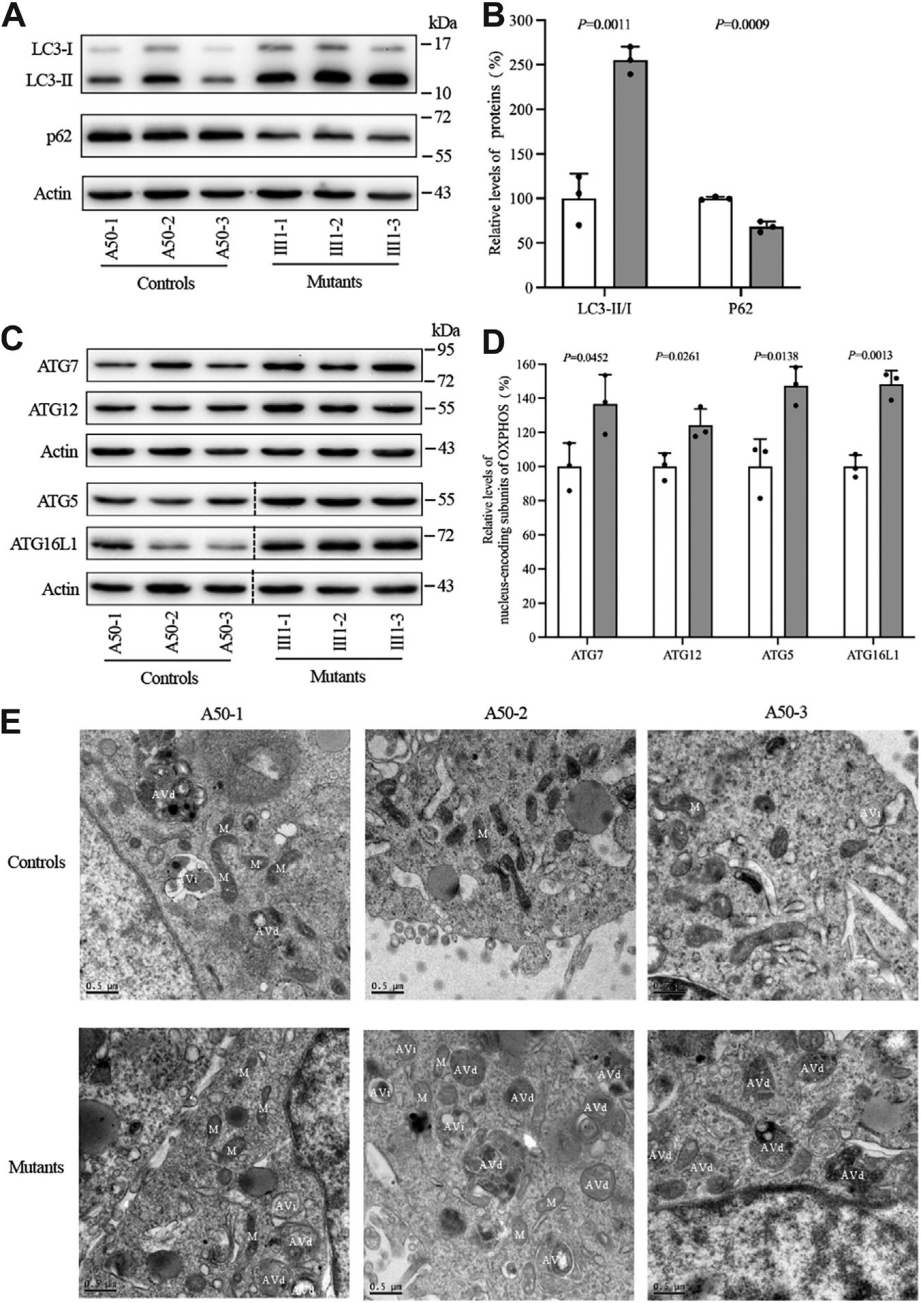
**Analysis of autophagy.***A*, Western blot analysis of autophagy markers LC3 and p62, employing actin as a loading control. *B*, quantification of LC3 and p62 levels in both mutant and control cells. *C*, Western blotting analysis of proteins associated with autophagosome formation and maturation (ATG7, ATG12, ATG5, ATG16L1) in six cell lines, with actin as a loading control. The splice borders were marked with *black* dashed lines. *D*, quantification of autophagosome formation and maturation-associated proteins. Each analysis was replicated across three independent experiments for robust assessment. *E*, TEM images providing visual insights into early autophagic vacuoles (Avi), degradative autophagic vacuoles (Avd), and mitochondria (M). Scale bar: 0.5 μm. Graph details and symbols are explained in Figure 1.

Increased mitochondrial fission by the m.15927G > A mutation might promote autophagy. To assess the effect of the m.15927G > A mutation on mitophagy, the mitophagy states in both control and mutant cells were evaluated *via* immunoblotting. Autophagy levels were evaluated using LC3 and p62 markers (20). LC3 transitions from a cytoplasmic form (LC3-I) to a cleaved, lipidated membrane-bound form (LC3-II) during autophagy, with LC3-II levels correlating to the number of autophagosomes. p62, also known as sequestosome 1 (SQSTM1), was a selective autophagy adaptor protein, often colocalizing with ubiquitinated aggregates in neurodegenerative conditions (21, 22). Cells carrying the m.15927G > A mutation exhibited increased LC3-II/I and decreased p62 levels compared to the control cells. Specifically, the average LC3-II/I and p62 levels in three mutant cell lines were 254.9% (*p* = 0.0011) and 68.1% (*p* = 0.0009), of the average values in control cell lines, respectively (Fig. 4, *A* and *B*). These results suggest an increase in autophagy in the cybrids harboring the m.15927G > A mutation.

Macroautophagy, commonly referred to as autophagy, involves autophagosomes that engulf cytoplasmic components and subsequently fuse with lysosomes. The autophagosome formation is regulated by about 20 genes, collectively known as core autophagy-related genes (ATG) (23). The expression levels of ATG7, ATG12, ATG5, and ATG16L1 were examined to elucidate the macroautophagy pathway. Figure 4, *C* and *D* demonstrated elevated ATG7, ATG12, ATG5, and ATG16L1 levels in cells bearing the m.15927G > A mutation compared to control cells. Specifically, the average levels of ATG7, ATG12, ATG5, and ATG16L1 in three mutants were 136.7% (*p* = 0.0452), 124.4% (*p* = 0.0261), 147.4% (*p* = 0.0138), and 148.2% (*p* = 0.0013), of those in controls, respectively.

To further investigate the process of autophagy, TEM was employed to examine autophagy and quantify autophagic accumulation in control and mutant cells. As shown in Figure 4*E*, the mutant cells showed a higher prevalence of matured late autophagic vacuoles than early autophagic vacuoles containing intact cytoplasm, as compared with those in the control cells. These findings indicated that the m.15927G > A mutation may impair autophagy, particularly affecting the formation of autolysosomes.

### Analysis of mitophagy

**Figure 5:**
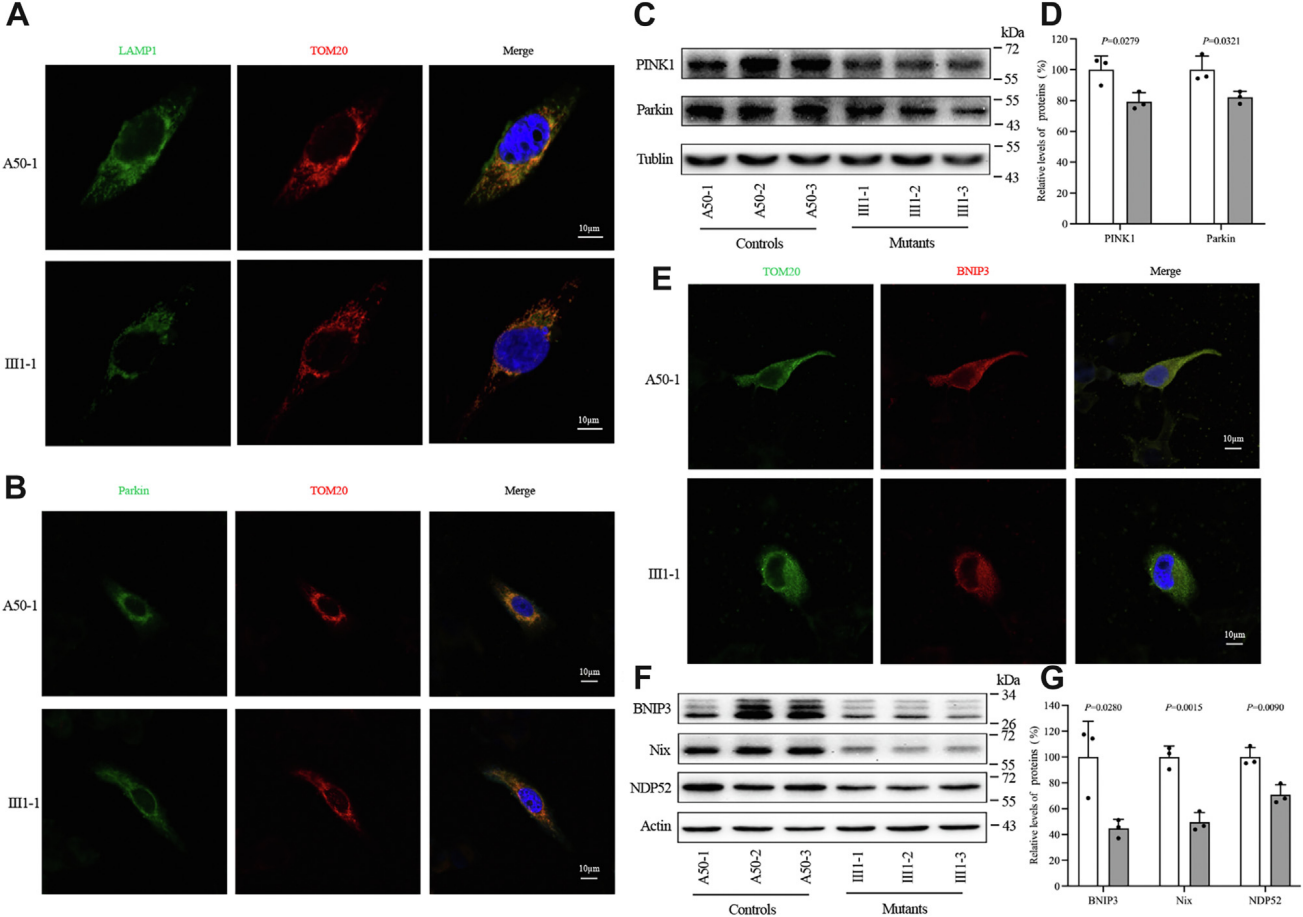
**Mitophagy analysis.***A, B, E*, immunofluorescence assays visualizing mitophagy in cybrids (III1-1 and A50-1) using LAMP1, Parkin, TOM20, and BNIP3 antibodies conjugated to Alex Fluor 488 (*green*) and Alex Fluor 555 (*red*) with DAPI-stained nuclei in *blue*. *C, F*, Western blot analysis assessing Parkin-dependent (*C*) and ubiquitination-independent (*F*) mitophagy proteins. *D, G*, Quantifying mitophagy proteins, specifically Parkin-dependent (*D*) and ubiquitination-independent (*G*), was undertaken for a comprehensive assessment. The repetition of each analysis was carried out over three independent experiments to ensure robust and reliable outcomes. Graph details and symbols are explained in Figure 1.

Mitophagy, a specialized type of autophagy, specifically targets damaged mitochondria for degradation by autophagosomes and lysosomes (24, 25, 26). This could be divided into two categories: ubiquitin-dependent or receptor-based mitophagy, which uses apoptosis-related proteins as mitophagy inhibitors or receptors, and ubiquitin-dependent mitophagy, including Parkin-dependent mitophagy (27). The effect of the m.15927G > A mutation on mitophagy was evaluated using immunofluorescence and immunoblotting assays. Figure 5*A* illustrates the immunofluorescence labeling results using antibodies targeting lysosome-associated membrane glycoprotein 1 (LAMP1) and TOM20. These findings suggested a decrease in mitophagy activity associated with the m.15927G > A mutation. Immunofluorescence and Western blot assays were employed to determine the mitophagic activities of mutant and control cell lines in order to further study the effects of the m.15927G > A mutation on mitophagy. These assays focused on proteins crucial for Parkin-dependent mitophagy (PINK1/Parkin pathway), as well as receptor-dependent mitophagy. In the context of acute mitochondrial dysfunction, the PINK1/Parkin pathway was activated, leading to the translocation of Parkin to the mitochondrial surface from the cytosol, thereby facilitating mitophagy (25, 26, 27). Pro-apoptotic proteins including BCL2 interacting protein 3 (BNIP3) and BNIP3-like (BNIP3L), which belong to the BCL2 family, or Nip3-like protein X (NIX) also modulated receptor-dependent mitophagy (28, 29). Immunofluorescence assays indicated a decrease in mitochondrial Parkin levels in mutant cybrids compared to control cybrids (Fig. 5*B*). Figure 5, *C* and *D* demonstrated diminished levels of PINK1 and Parkin in mutant cybrids carrying the m.15927G > A mutation compared to control cybrids. Specifically, the mean levels of Parkin and PINK1 in the three mutants were 79.4% (*p* = 0.0279), and 82% (*p* = 0.0321) respectively, compared to the average in the three controls, indicating reduced mitophagy attributed to the mutation. As demonstrated in Figure 5*E*, BNIP3 levels were markedly reduced in mutant cybrids. Additionally, the levels of BNIP3, NIX, and NDP52 in cybrids with the m.15927G > A mutation were 44.6%, 49.5%, and 70.8% of the average of three controls, respectively (Fig. 5, *F* and *G*). Based on these results, it can be concluded that the m.15927G > A mutation downregulates the Parkin-mediated and receptor-dependent mitophagy.

### Promoting intrinsic apoptosis

**Figure 6:**
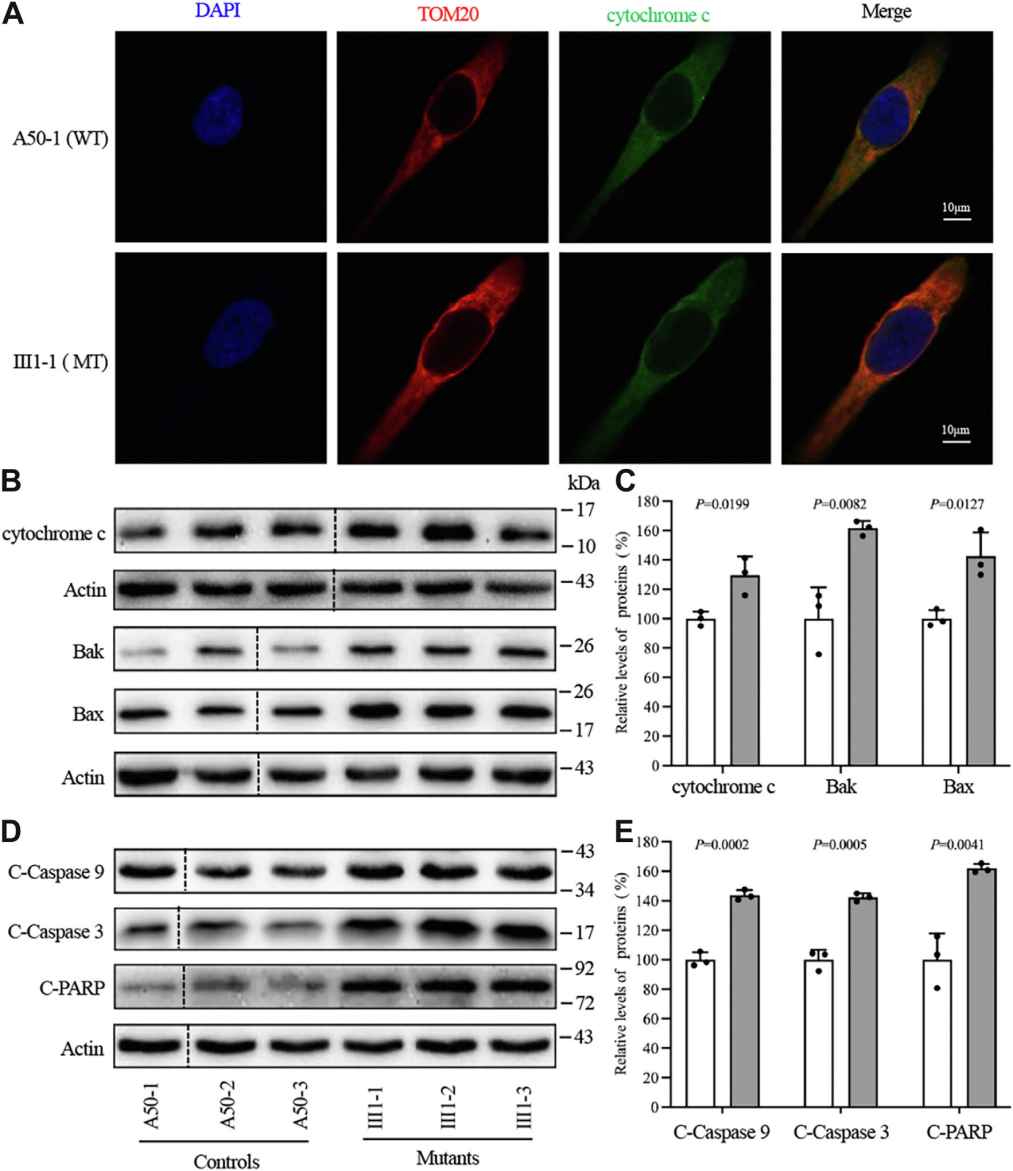
**Apoptosis assays.***A*, immunofluorescence analysis displaying the distribution of cytochrome c in mutant III1-1 and wild-type A50 to 1 cybrids, using antibodies against cytochrome c (green) and TOM20 (red), with DAPI stained nuclei in *blue*, analyzed *via* confocal microscopy. *B*, Western blot analysis for apoptotic markers cytochrome c, BAK, and BAX, with actin as a loading control. The splice borders were marked with black dashed lines. *C*, quantitative evaluation of cytochrome c, BAK, and BAX levels. *D*, conducting Western blot analysis on apoptosis-activated proteins (cleaved caspases 9 and 3, and PARP), utilizing actin as a loading control. The splice borders were marked with *black* dashed lines. *E*, quantification of these apoptosis-activated proteins. Each experiment was replicated three times. Graph details and symbols are explained in Figure 1.

Mitochondrial dysfunctions and increased ROS production, commonly seen in LHON-related mtDNA mutations, were frequently associated with apoptosis mediated by mitochondria (30, 31). By analyzing the apoptotic status of cybrids, the impact of the m.15927G > A mutation on apoptotic processes was assessed. This was achieved through immunocytostaining, confocal microscopy, and Western blotting analysis. As depicted in Figure 6*A*, immunofluorescence patterns show cybrids doubly labeled with antibodies targeting cytochrome c and TOM20, a nuclear-encoded mitochondrial inner membrane protein. The cytochrome c, BAK, and BAX levels in mutant cell lines were increased by 29.6%, 61.6%, and 42.5%, respectively, compared to control cell lines (Fig. 6, *B* and *C*). Western blot analysis was employed to quantify proteins linked with apoptosis in order to further investigate the effects of the m.15927G > A mutation on apoptosis. The analysis indicated that the average levels of cleaved caspase 9, 3, and PARP in mutant cell lines were 143.8%, 142.3%, and 161.9% of those in control cell lines, respectively (*p* = 0.0002, 0.005, and 0.0041) (Fig. 6, *D* and *E*).

## Discussion

The regulatory mechanism through which mitochondrial tRNA mutations impact the expression of nuclear genes, crucial for the maintenance of healthy mitochondrial cellular function and populations, remains poorly understood. The deficient t^6^A modification of mitochondrial tRNA^Thr^ caused by m.15927G > A led to the instability of the anticodon loop structure, abnormal in tRNA binding to the A-site codon and impaired the efficiency and accuracy of translation, thereby affected the synthesis of mtDNA-encoding CI and CIII subunits, especially in the fidelity and efficiency (17, 18, 32, 33, 34). As a consequence, this disruption in tRNA^Thr^ metabolism resulted in respiratory deficiency, reduced membrane potential, and increased oxidative stress (17, 18, 35). Particularly, the altered quality and quantity of these mtDNA encoding proteins may result in imbalances between the increased levels of *de novo* protein synthesis and decreased folding capacity for the mtDNA- and nucleus-encoded OXPHOS subunits (36). In this investigation, cybrids with the m.15927G > A mutation demonstrated reduced levels of nucleus-encoded subunits of CI and CIII, specifically NDUFA9 and UQCRC2, while the levels of other subunits such as SDHA, COX IV, and ATP5A remained unaffected. Notably, CI and CIII had aberrant assembly and instability as a result of these deficiencies, which reduced their activity. Therefore, OXPHOS-related nuclear gene expression was dysregulated by the m.15927G > A mutation, affecting the genes' stability, activity, and assembly. Meanwhile, a coronary artery disease-associated m.15927G > A mutation led to variable decreases in the levels of ND1, ND3, ND4, ND5, CO2, CYTB, ATP6, and ATP8, whereas ND6 with lower threonine codons and nuclear genes encoded subunits NDUFB8, SDHB, UQCRC2 and ATP5A in mutant cybrid cell lines were comparable with those in control cells (17, 35). We suggested that the different results might be caused by the different nuclear backgrounds (one fused with HUVECs-less-mtDNA lines and the other fused with mtDNA-less human ρ°206 cells, derived from the 143B.TK− cell line), and mtDNA haplogroups (mtDNA haplogroup B5 and haplogroup G2a, respectively). Cybrids carrying the m.4435A > G mutation displayed various reductions in the nuclear-encoded OXPHOS subunits of CI, CIII, CIV, and CV. These deficiencies gave rise to the abnormal assembly and instability of complexes I, III, IV, and V as well as intact supercomplexes observed in cell lines bearing the m.4435A > G mutation. The m.4435A > G mutation dysregulated the expression of nucleus-encoding OXPHOS subunits and thereby impaired the assembly, stability, and activity of OXPHOS (37).

The m.15927G > A mutation causes OXPHOS dysfunction, which makes mitochondria integrity undergo continual fusion and fission. This process repairs damaged OXPHOS components, segregates damaged mitochondria through fission, facilitates material exchange between healthy mitochondria through fusion, and ultimately eliminates damaged mitochondria through mitophagy (38). Our investigations revealed structural abnormalities in mitochondria, such as increased fragmentation and a decreased formation of the elongated network, alongside an imbalance in mitochondrial dynamics-related proteins in cells bearing the m.15927G > A mutation. These mutant cells demonstrated a significant increase in the expression of mitochondrial fission genes (DRP1 and MFF), but no significant changes in that of three fusion-related proteins (OPA1, MFN1, and MFN2). These findings manifest that the m.15927G > A mutation disrupts mitochondrial dynamics by enhancing fission. Meanwhile, the m.4435A > G mutation dysregulated mitochondrial dynamics through promoting fission and reducing fusion (37).

The m.15927G > A mutation disrupts the equilibrium of mitochondrial dynamics and alters membrane potential, thereby influencing the process of mitophagy, a specialized form of autophagy tasked with eliminating dysfunctional mitochondria (39). Cells harboring the m.15927G > A mutation showed a significant accumulation of advanced autophagic vacuoles and increased levels of the proteins LAMP1 and LC3. An increase in LC3 levels and a decrease in p62 levels in cells bearing m.15927G > A mutation indicate that the process of autophagy, including the initiation phase, was impaired. Furthermore, it also influences the formation and maturation of autophagosomes, as shown by increased ATG5, ATG7, ATG16L1, and ATG12-ATG5 levels (27, 39). This altered autophagic response is consistent with findings in cells with mitochondrial tRNA mutations like tRNA^Met^ 4435A > G, tRNA^Ala^ 5587T > C and tRNA^Ile^ 4295A > G (37, 40, 41), suggesting that mutations in mitochondrial tRNA might upregulate the autophagy pathway by modifying the quality and quantity of the 13 mitochondrial proteins. Pathologically increased autophagy and mitophagy also observed in cells from LHON-affected patients using patient-derived fibroblasts, induced pluripotent stem cell (iPSC)-derived neuronal cell lines, peripheral blood mononuclear cells (PBMCs) (42). In contrast, specific mutations like m.14484T > C and m.3460G > A, which impact the ND1 or ND6 proteins, appear to suppress the autophagy process (43, 44). A healthy mitochondrial population within cells is maintained *via* mitophagy, which is essential for eliminating damaged mitochondria (45). The regulatory mechanisms of mitophagy are broadly categorized into ubiquitin-dependent and independent pathways (46). Mitochondrial depolarization can activate the PINK1 pathway, recruiting Parkin to the mitochondria for clearance, while extensive mitochondrial fission might trigger a form of mitophagy that is independent of depolarization (27). Notably, in cells harboring the m.15927G > A mutation, Parkin-mediated mitophagy was downregulated, as indicated by significantly decreased levels of PINK1 and Parkin. This observation aligns with previous findings of pathologically decreased Parkin-dependent mitophagy in individuals affected by LHON (43, 44). Furthermore, the m.15927G > A mutation appears to negatively affect receptor-dependent mitophagy, as evidenced by significantly reduced levels of and BNIP3, which function as pro-apoptotic proteins and mitophagy receptors, in cells harboring this mutation (28, 46). Analysis of BNIP3 and BNIP3L/Nix expression in cybrid cell lines harboring two LHON-associated 11778G > A and 3460G > A LHON mutations revealed that BNIP3 protein level is decreased in LHON cells compared to controls (47). These findings suggest downregulation of Parkin-mediated mitophagy, as well as receptor-dependent mitophagy in cell lines harboring the m.15927G > A mutation.

Mitochondrial dysfunctions and increased production of ROS, commonly associated with LHON-related mtDNA mutations, have been shown to influence apoptotic cell death and mitophagy (30, 31, 48, 49, 50). In this study, mitochondrial dysfunctions resulting from the m.15927G > A mutation were observed to promote the apoptotic process. Western blot analysis and immunocytostaining assays revealed increased cytochrome c release into the cytosol in mutant cybrids compared to control cybrids. This observation was corroborated by elevated expression levels of BAX and BAK, pivotal regulators of apoptosis, as well as improved levels of activated apoptotic proteins such as cleaved caspases 9, 3, and PARP, observed in the mutant cybrids in contrast to the controls (51, 52). Cytochrome c release from mitochondria affects caspase activation, which afterward initiates apoptosis (53, 54). The reduction in anti-apoptotic proteins BNIP3L/NIX and Bcl-xL expression further confirms the influence of the m.15927G > A mutation on intrinsic apoptosis (20, 55, 56). These outcomes highlight that the mitochondrial dysfunction arising from the m.15927G > A mutation could induce perturbations in mitochondrial dynamics, culminating in the preferential degradation of compromised mitochondria and triggering the intrinsic apoptosis pathway.

**Figure 7:**
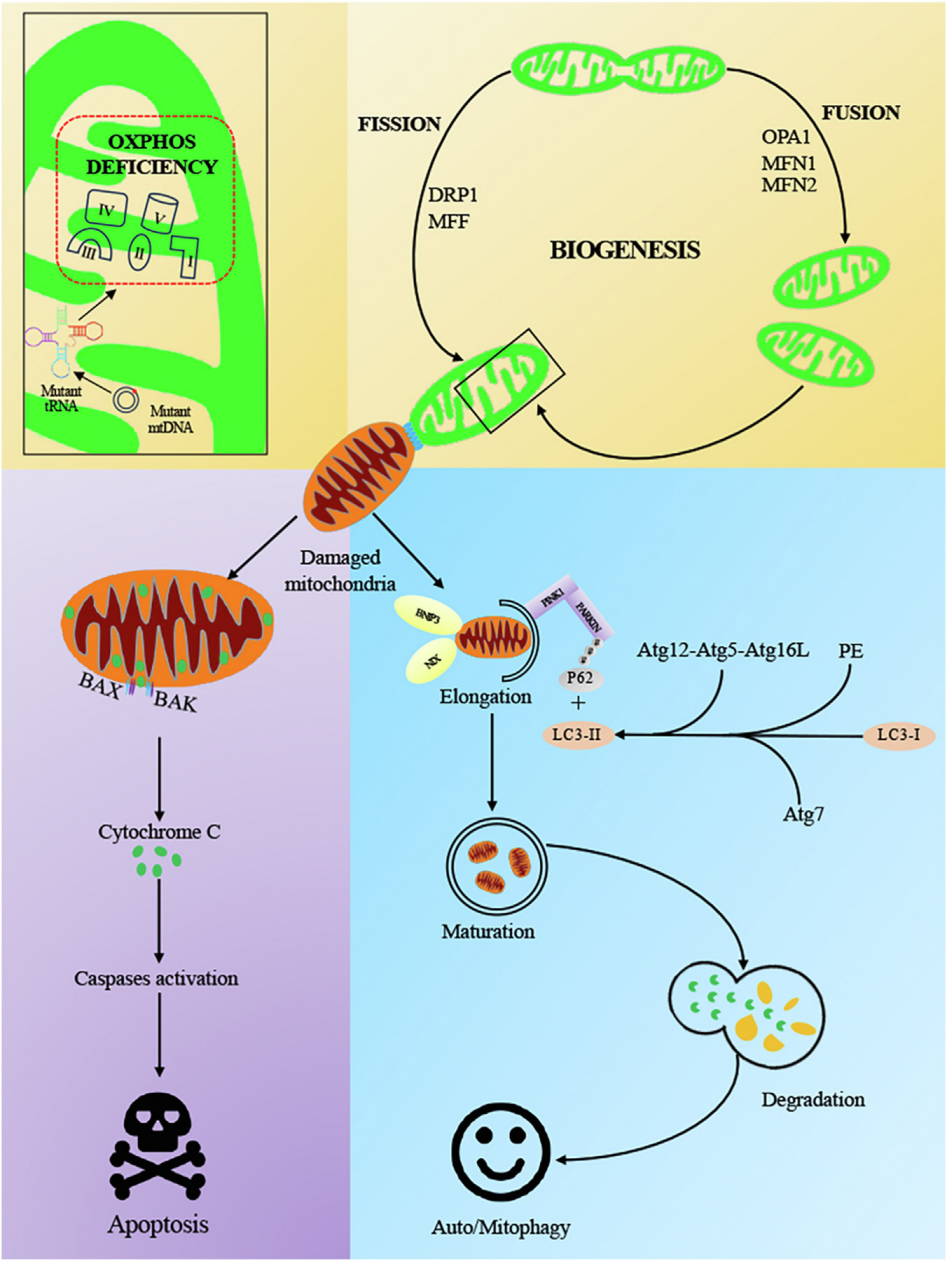
**Model for m.15927G > A mutation leading to LHON.** The LHON-associated m.15927G > A mutation results in abnormal OXPHOS biogenesis, aberrant mitochondrial quality control, increased autophagy, decreased mitophagy and elevated apoptotic cell death.

In summary, the present investigation elucidated that the homoplasmic m.15927G > A mutation exerts an impact on the assembly and biogenesis of OXPHOS complexes by disrupting the regulatory balance in the expression of OXPHOS subunits encoded by nuclear genes. These mitochondrial dysfunctions induce an imbalance in mitochondrial dynamics *via* fission. They also increased mitophagy through Parkin-dependent mitophagy and receptor-dependent mitophagy, ultimately triggering intrinsic apoptotic processes. These results highlight the critical involvement of the m.15927G > A mutation in the pathogenesis of LHON, impacting the signalling pathways linked to the biogenesis of OXPHOS, mitochondrial quality control, autophagy, and apoptosis (Fig. 7). Therefore, our findings may provide new insights into the pathophysiological development of LHON. However, the tissue-specific effect of mitochondrial dysfunction on the RGCs deficiencies for the expression of LHON needs to be identified by iPSCs-derived RGCs from patients carrying m.15927G > A mutation.

## Experimental procedures

### Cell lines and cultivation conditions

This study was in compliance with the tenets of the Declaration of Helsinki. Informed consent, blood samples, and clinical evaluations were obtained from all participating family members under protocols approved by the ethics committee of Wenzhou Medical University. Cybrids were constructed by fusing mtDNA-less human ρ0206 cells with enucleated cells derived from lymphoblastoid cells of an affected individual in Chinese family WZ600 (III1) carrying m.15927G > A homoplasmic mutation and an unrelated genetically matched control sharing the same mtDNA haplogroup (A50) (57, 58). The cybrid clones were isolated by growing the fusion mixtures in a selective DMEM medium, containing BrdU and G418, but lacking uridine. Discrette clones should be detectable 10 to 14 days after selection. Several healthy cybrid clones were isolated, and subsequently analyzed for the presence and level of the m.15927G > A mutation. The results confirmed the absence of the mtDNA mutation in the control clones and their presence in homoplasmy in all cybrids derived from the mutant cell lines (Fig. S1). These resultant cybrids were identified and meticulously cultured in Dulbecco’s modified Eagle’s medium (DMEM), undergoing supplementation with 10% fetal bovine serum (FBS) as a previously outlined protocol (18). Three cybrids derived from each donor cell line with similar mtDNA copy numbers were used for the biochemical characterization described below.

### RT-qPCR

TRIzol reagent (Beyotime) was utilized to isolate total RNA of cybrids. RNA quality was assessed by NanoDrop One (Thermo Fisher Scientific). RNAs were used for RT-qPCR following the general manufacturer’s recommendations with some modifications (43). Briefly, RNAs were reverse transcribed into cDNAs with random primer, and cDNAs were further amplified using specific primers (Table S1). The target gene expression was quantified with 2^-ΔΔCt^ method based on the Actin normalized data.

### SDS-PAGE and immunoblotting

In preparation for immunoblotting analysis, cellular lysis was executed using ice-cold RIPA lysis buffer enriched with a freshly incorporated protease inhibitor cocktail (Beyotime). Following a meticulous 30-min incubation on ice, the lysates underwent a centrifugation process at 14,000 rpm under chilled conditions of 4 °C for a duration of 20 min. The resulting supernatant, carefully harvested, was designated for subsequent protein concentration analysis and stored at either −20 °C or −80 °C for optimal preservation. Pierce BCA Protein Assay Kit was used to quantify protein concentration. Each individual sample underwent a thorough amalgamation with 5 × SDS loading buffer, followed by a controlled heating process at 95 °C for 5 min. Subsequently, 20 μg of proteins were loaded onto the SDS-PAGE. After that, PVDF membranes were used to transfer the proteins that had been separated on the gels for hybridization. Cell Signalling Technology (CST) provided the antibodies used in this work, [DRP1 (5391), MFF (84850), OPA1 (80471), MFN1 (14739), MFN2 (11925), LAMP1 (9091T), LC3 (12741), p62 (8025), ATG16L1 (8089), ATG12 (4180), ATG7 (8558), ATG5 (12994), PINK1 (6946), Parkin (4211), NIX (12396), NDP52 (60732), BAK (12105), BAX (5023), Cleaved PARP (5625), Cleaved Caspase 3 (9664), Cleaved Caspase 9 (52873)], and abcam [cytochrome c (ab13575), NDUFA9 (ab14713), SDHA (ab14715), UQCRC2 (ab14745), COX IV (ab14744), ATP5A (ab14748)]. Following this, the membranes were incubated with the corresponding host secondary antibodies [anti-mouse IgG, HRP-linked Antibody (7076) or anti-rabbit IgG, HRP-linked Antibody (7074) (CST)], and protein signals were detected using the FluorChem Q System (ProteinSimple). Quantification of density in each band was conducted using ImageJ software, and the values were normalized to the corresponding loading control.

### BN-PAGE and in-gel activity assay

The control and impacted cybrids' mitochondrial proteins were subjected to in-gel activity assays and blue native polyacrylamide gel electrophoresis (BN-PAGE) using previously described protocols (19, 59). As per procedure, a gradient gel with a range of 3% - 11% was employed to separate 30 μg of mitochondrial proteins from each sample. Post-electrophoresis, the proteins underwent immunostaining using primary antibodies sourced from Abcam. These included NDUFA9 (ab14713) for complex I (CI), SDHA (ab14715) for complex II (CII), UQCRC2 (ab14745) for complex III (CIII), COX IV (ab14744) for complex IV (CIV), and ATP5A (ab14748) for complex V (CV). The primary antibodies were then conjugated with secondary HRP-linked goat anti-rabbit or goat anti-mouse IgG antibodies (CST), and protein signals were detected as described previously (43).

The in-gel activity assay was executed following established procedures (59). In brief, BN-PAGE gels underwent immersion in ice-cold water and enzymatic reactions were halted using 10% acetic acid. The gels were incubated with 10 ml freshly prepared staining solutions at room temperature [NADH dehydrogenase staining solution for Complex I: 0.1 M Tris-HCl pH 7.4, 0.1% (w/v) nitroblue tetrazolium (NBT), 0.14 mM NADH. Succinate dehydrogenase staining solution for Complex II: 50 mM phosphate buffer pH 7.4, 84 mM succinic acid, 0.2 mM phen-azine methosulfate, 0.2% (w/v) nitroblue tetrazolium. Cytochrome c reductase staining solution for Complex III: Pierce 1-Step TMB-Blotting Substrate solution (Pierce, Rockford, IL, USA). Cytochrome c oxidase staining solution for Complex IV: 10 mM phosphate buffer pH 7.4, 0.1% (w/v) 3,3′-diaminobenzidine (DAB), and 16 mM cytochrome c. ATPase staining solution for Complex V: 35 mM Tris-HCl pH 7.4, 270 mM glycine, 14 mM MgSO_4_, 5 mM ATP, and 0.2% Pb(NO_3_)_2_]. Staining takes 30 to 45 min for NADH dehydrogenase, up to several hours for succinate dehydrogenase, minimum of 6 h for cytochrome c reductase, approximately 2 h for cytochrome c oxidase, and 3 h to overnight incubation for ATPase. Following corresponding activity stains, wash gels extensively in water. Scan or take pictures of the stained gels immediately after activity staining.

### Immunofluorescence analysis

We cultivate cells on glass coverslips (Thermo Fisher Scientific), and subsequently fixed with 4% (w/v) formaldehyde (Sigma) for a duration of 10 min at room temperature. Following fixation, a permeabilization step was undertaken utilizing 0.2% Triton X-100 (Sigma) for 10 min, followed by a blocking procedure with 5% BSA for a period of 1 h. Overnight 4 °C incubation was carried out with primary antibodies from Abcam [TOM20 (ab56783), cytochrome c (ab13575)], Proteintech [TOM20 (11802-1-AP)], and CST [DRP1 (5391), LAMP1 (9091T), Parkin (4211), BNIP3 (44060)]. After undergoing three consecutive 5-min PBS washes at room temperature, the samples were subjected to a 1-h incubation at room temperature with secondary fluorescent antibodies, namely Alexa Fluor 488 goat anti-mouse IgG (H + L) (Beyotime) and Alexa Fluor 555 goat anti-rabbit IgG (H + L) (Beyotime). After this incubation, the cells were meticulously stained with DAPI (Beyotime) for a precise duration of 15 min and then carefully mounted using Fluoromount (Thermo Fisher Scientific). The acquisition of images was accomplished utilizing a confocal microscope (LSM880) from Carl Zeiss that was headquartered in Germany equipped with three lasers (Ex/Em = 358/461 nm, 488/520 nm, and 555/570 nm).

### Transmission electron microscopy (TEM)

The specimens were fixed by immersing them in a solution comprising 2.5% glutaraldehyde in 0.1 M phosphate buffer (pH 7.0) for a period exceeding 4 h. Thereafter, post-fixation stage was conducted, utilizing 1% osmium tetroxide (OsO4) within the same buffer, sustained for a 2-h duration. Each sample was washed three times in 0.1 M phosphate buffer (pH 7.0) after fixation, lasting 15 min for each wash. A sequentially graded series of ethanol concentrations, spanning 30%, 50%, 70%, 80%, 90%, 95%, and culminating at 100%, was utilized, with a 20-min ethanol soak between each to achieve optimal dehydration. This was succeeded by a comprehensive 20-min immersion in pure acetone. In the subsequent steps of the procedure, the samples were immersed sequentially in a 1:1 mixture of pure acetone and spur resin for 1 h, followed by a 1:3 mixture for 3 h. Finally, they were immersed in pure spur resin overnight. All these steps were undertaken at room temperature. Embedding into spur resin within specifically designated Eppendorf tubes was carried out, with a subsequent heating process at 70 °C sustained for an extensive period exceeding 9 h. Further processing involved the preparation of ultrathin sections utilizing a LEICA EM UC7 ultramicrotome. Every staining session lasted 5 to 10 min, and the staining agents used were 2% alkaline lead citrate and uranyl acetate. The culmination of this intricate methodology involved the examination of the specimens through a high-resolution transmission electron microscope from Hitachi (H-7650).

### Quantification and statistical analysis

The data were attained from three biologically independent experiments. All statistical tests were carried out using GraphPad Prism 9.0 software. For comparison between two groups, the Student's *t* test was employed, whereas analyses involving multiple groups underwent ordinary one-way ANOVA with Dunnett's multiple comparison test. All quantitative data were shown as the mean ± SEM. Differences were considered statistically significant at *p* < 0.05.

## Data availability

The authors confirm that the data supporting the findings of this study are available within the article and its supplementary materials. For any additional information, please contact the corresponding author.

## Supporting information

This article contains supporting information.

## Conflict of interest

The authors declare that they have no conflicts of interest with the contents of this article.

## Figures and Tables

**Supporting Figure 1:**