# Supplementary material for: Defective post-transcriptional modification of tRNA disrupts mitochondrial homeostasis in Leber’s hereditary optic neuropathy
Source: J Biol Chem. 2024 Aug 28;300(9):107728. doi: 10.1016/j.jbc.2024.107728 (PMC11421333; doi:10.1016/j.jbc.2024.107728)
Supplement: Supporting Table 1 [file mmc1.docx]

Supplementary Table 1. Primers of RT-qPCR

| Gene | Forward primer | Reverse primer |
| --- | --- | --- |
| NDUFA9 | AAgCCATgTggATCCTgTgA | TCCTTTCTCCCACTTgCTgT |
| SDHA | TggACCTggTTgTCTTTggT | gAgTCgCAgTTCCgATgTTC |
| UQCRC2 | gCAACTCAgCAgCCATTTgA | TTgTTCTTggCAgCTTggAC |
| COX IV | TggTgCTgAggAAAggTggAg | AACTggAgCCggTACAAgg |
| ATP5A | TggACCTggTTgTCTTTggT | gAgTCgCAgTTCCgATgTTC |
| Actin | gATCATTgCTCCTCCTgAgC | ACATCTGCTggAAggTggAC |
